# *N*-(4-Chloro­phen­yl)-4-meth­oxy­benzamide

**DOI:** 10.1107/S1600536812041384

**Published:** 2012-10-06

**Authors:** Rajni Kant, Seema Sahi, Vivek K. Gupta, Kamini Kapoor, Satya Paul

**Affiliations:** a X-ray Crystallography Laboratory, Post-Graduate Department of Physics & Electronics, University of Jammu, Jammu Tawi 180 006, India; b Post-Graduate Department of Chemistry, University of Jammu, Jammu Tawi 180 006, India

## Abstract

In the title compound, C_14_H_12_ClNO_2_, the mean plane through the amide group [–N—C=O–] forms dihedral angles of 27.55 (8) and 31.94 (7)° with the meth­oxy- and chloro-substituted benzene rings, respectively. The dihedral angle between the benzene rings is 59.24 (4)°. In the crystal, N—H⋯O and weak C—H⋯O hydrogen bonds link the mol­ecules into chains along the *a* axis.

## Related literature

For the biological activity of amides, see: Chen *et al.* (2011 ▶); El Rayes *et al.* (2008 ▶); Regiec *et al.* (2006 ▶); Kuroda *et al.* (2006 ▶). For related structures, see: Gowda *et al.* (2008 ▶); Saeed *et al.* (2008 ▶).
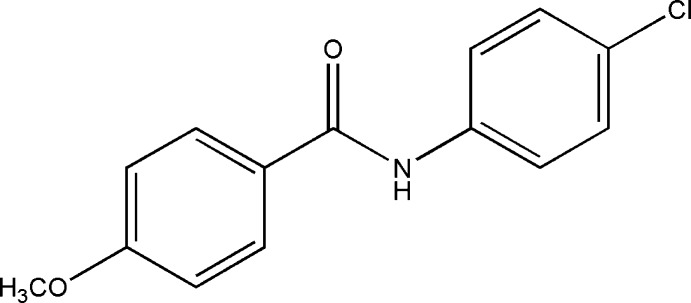


## Experimental

### 

#### Crystal data

C_14_H_12_ClNO_2_
*M* *_r_* = 261.70
Triclinic, 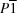

*a* = 5.4394 (2) Å
*b* = 7.7754 (3) Å
*c* = 14.9262 (6) Å
α = 78.759 (3)°
β = 80.712 (3)°
γ = 88.821 (3)°
*V* = 611.01 (4) Å^3^
*Z* = 2
Mo *K*α radiation
μ = 0.31 mm^−1^
*T* = 293 K
0.3 × 0.2 × 0.2 mm

#### Data collection

Oxford Diffraction Xcalibur Sapphire3 diffractometer
Absorption correction: multi-scan (*CrysAlis PRO*; Oxford Diffraction, 2010 ▶) *T*_min_ = 0.952, *T*_max_ = 1.000
14438 measured reflections
2407 independent reflections
1997 reflections with *I* > 2σ(*I*)
*R*_int_ = 0.035

#### Refinement

*R*[*F* ^2^ > 2σ(*F* ^2^)] = 0.039
*wR*(*F* ^2^) = 0.100
*S* = 1.03
2407 reflections
168 parameters
H atoms treated by a mixture of independent and constrained refinement
Δρ_max_ = 0.17 e Å^−3^
Δρ_min_ = −0.32 e Å^−3^

### 

Data collection: *CrysAlis PRO* (Oxford Diffraction, 2010 ▶); cell refinement: *CrysAlis PRO*; data reduction: *CrysAlis PRO*; program(s) used to solve structure: *SHELXS97* (Sheldrick, 2008 ▶); program(s) used to refine structure: *SHELXL97* (Sheldrick, 2008 ▶); molecular graphics: *ORTEP-3* (Farrugia, 1997 ▶); software used to prepare material for publication: *PLATON* (Spek, 2009 ▶).

## Supplementary Material

Crystal structure: contains datablock(s) I, New_Global_Publ_Block. DOI: 10.1107/S1600536812041384/lh5539sup1.cif

Structure factors: contains datablock(s) I. DOI: 10.1107/S1600536812041384/lh5539Isup2.hkl

Supplementary material file. DOI: 10.1107/S1600536812041384/lh5539Isup3.cml

Additional supplementary materials:  crystallographic information; 3D view; checkCIF report

## Figures and Tables

**Table 1:** Hydrogen-bond geometry (Å, °)

*D*—H⋯*A*	*D*—H	H⋯*A*	*D*⋯*A*	*D*—H⋯*A*
N1—H1⋯O1^i^	0.83 (4)	2.47 (2)	3.222 (2)	151 (2)
C14—H14⋯O1^i^	0.93	2.56	3.251 (2)	131

Symmetry code: (i) 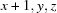.

**Fig. 1.**
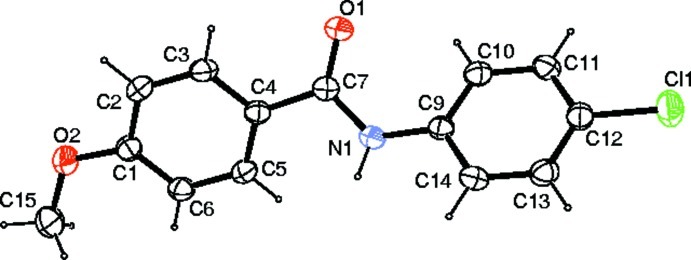
The molecular structure of (I) shown with 40% probability ellipsoids. H atoms are shown as small spheres of arbitrary radii.

**Fig. 2.**
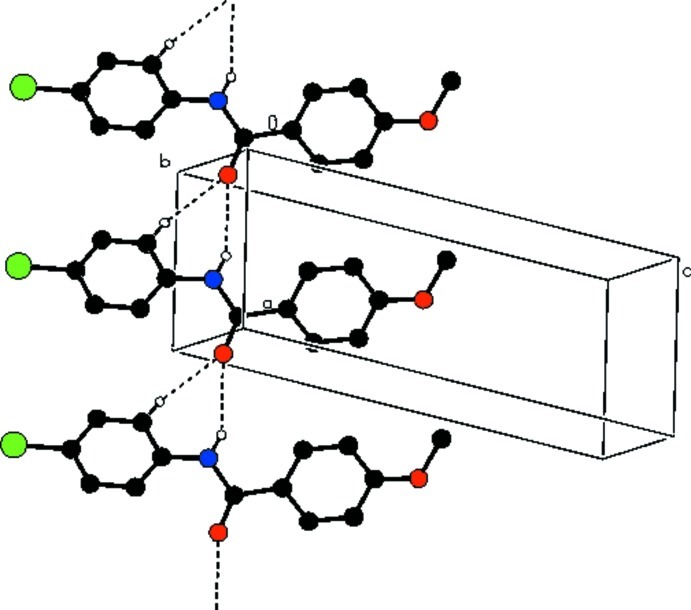
The packing arrangement of molecules with dashed lines to show intermolecular N—H···O and weak C—H···O hydrogen bonds. Only H atoms involved in hydrogen bonds are shown.

## supplementary crystallographic information

### Comment

Aromatic amides have been utilized as versatile fragments to construct frameworks that have numerous functions such as molecular recognition, conformational switching, and biological activities which include *in vitro* xanthine oxidase (XO), tyrosinase and melanin production inhibitory activity. Further, benzanilides and their metal complexes have also been known for their marked biological activities such as antifungal,antibacterial and genotoxic effect (Chen *et al.*, 2011). Globally, multidrug-resistant bacteria are a major health problem leading to severe consequences; anilides have shown antimycobacterial activity against classical mycobacterium tuberculosis (El Rayes *et al.*, 2008), thiobenzanilides also belong to a group of biologically active compounds possessing antimicobacterial activities. In addition, N-substituted carboxamide isothiazoles (Regiec *et al.*, 2006) produced a remarkable immunotropic, antiviral and anti-inflammatory activities, *N*-acylamino benzamides (Kuroda *et al.*, 2006), constitute an important class of potent insecticides.

The molecular structure of the title compound (I) is shown in Fig. 1. All bond lengths and angles are normal and correspond to those observed in the related structures (Gowda *et al.*, 2008; Saeed *et al.*, 2008). The amide group [–N—C═O–] forms dihedral angles of 27.55 (8) and 31.94 (7)° with methoxy-substituted benzene [C1-C6] and chloro-substitued benzene [C9-C14] rings, respectively. The two benzene rings are twisted by 59.24 (4)° with respect to each other. In the crystal, N1—H1···O1^i^ and C14—H14A···O1^i^ hydrogen bonds link the molecules into chains along the *a* axis (Fig. 2) (Table 1).

### Experimental

To a mixture of 4-chloroaniline (0.127 g, 1 mmol) and aq. sodium hydroxide solution (10%, 20 ml) in a round bottom flask (50 ml), 4-methoxybenzoyl chloride (0.170 g, 1 mmol) was added in portions during stirring at room temperature. After the complete addition of 4-methoxybenzoyl chloride, the reaction mixture was further stirred for 30 minutes and then poured into ice-cold water (25 ml). It was further stirred for 10 min. and filtered. Finally, the product was obtained after drying followed by crystallization from ethanol (0.224 g, 86%) to give X-ray quality crystals.

### Refinement

The H atom bonded to the N atom was located in a difference map and refined independently with an isotropic displacement parameter. Other H atoms were positioned geometrically and were treated as riding on their parent C atoms, with C—H distances of 0.93–0.96 Å and with with *U*_iso_(H) = 1.2*U*_eq_(C) or 1.5*U*_eq_(methyl C).

### Crystal data

**Table:**

C_14_H_12_ClNO_2_	*Z* = 2
*M**_r_* = 261.70	*F*(000) = 272
Triclinic, *P*1	*D*_x_ = 1.422 Mg m^−^^3^
Hall symbol: -P 1	Mo *K*α radiation, λ = 0.71073 Å
*a* = 5.4394 (2) Å	Cell parameters from 6498 reflections
*b* = 7.7754 (3) Å	θ = 3.5–29.0°
*c* = 14.9262 (6) Å	µ = 0.31 mm^−^^1^
α = 78.759 (3)°	*T* = 293 K
β = 80.712 (3)°	Rectangular, white
γ = 88.821 (3)°	0.3 × 0.2 × 0.2 mm
*V* = 611.01 (4) Å^3^	

### Data collection

**Table:**

Oxford Diffraction Xcalibur Sapphire3 diffractometer	2407 independent reflections
Radiation source: fine-focus sealed tube	1997 reflections with *I* > 2σ(*I*)
Graphite monochromator	*R*_int_ = 0.035
Detector resolution: 16.1049 pixels mm^-1^	θ_max_ = 26.0°, θ_min_ = 3.5°
ω scan	*h* = −6→6
Absorption correction: multi-scan (*CrysAlis PRO*; Oxford Diffraction, 2010)	*k* = −9→9
*T*_min_ = 0.952, *T*_max_ = 1.000	*l* = −18→18
14438 measured reflections	

### Refinement

**Table:**

Refinement on *F*^2^	Primary atom site location: structure-invariant direct methods
Least-squares matrix: full	Secondary atom site location: difference Fourier map
*R*[*F*^2^ > 2σ(*F*^2^)] = 0.039	Hydrogen site location: inferred from neighbouring sites
*wR*(*F*^2^) = 0.100	H atoms treated by a mixture of independent and constrained refinement
*S* = 1.03	*w* = 1/[σ^2^(*F*_o_^2^) + (0.0427*P*)^2^ + 0.2321*P*] where *P* = (*F*_o_^2^ + 2*F*_c_^2^)/3
2407 reflections	(Δ/σ)_max_ < 0.001
168 parameters	Δρ_max_ = 0.17 e Å^−^^3^
0 restraints	Δρ_min_ = −0.32 e Å^−^^3^

### Special details

**Table:**

Experimental. *CrysAlis PRO*, Oxford Diffraction Ltd., Version 1.171.34.40 (release 27–08-2010 CrysAlis171. NET) (compiled Aug 27 2010,11:50:40) Empirical absorption correction using spherical harmonics, implemented in SCALE3 ABSPACK scaling algorithm.
Geometry. All e.s.d.'s (except the e.s.d. in the dihedral angle between two l.s. planes) are estimated using the full covariance matrix. The cell e.s.d.'s are taken into account individually in the estimation of e.s.d.'s in distances, angles and torsion angles; correlations between e.s.d.'s in cell parameters are only used when they are defined by crystal symmetry. An approximate (isotropic) treatment of cell e.s.d.'s is used for estimating e.s.d.'s involving l.s. planes.
Refinement. Refinement of *F*^2^ against ALL reflections. The weighted *R*-factor *wR* and goodness of fit *S* are based on *F*^2^, conventional *R*-factors *R* are based on *F*, with *F* set to zero for negative *F*^2^. The threshold expression of *F*^2^ > σ(*F*^2^) is used only for calculating *R*-factors(gt) *etc*. and is not relevant to the choice of reflections for refinement. *R*-factors based on *F*^2^ are statistically about twice as large as those based on *F*, and *R*- factors based on ALL data will be even larger.

### Fractional atomic coordinates and isotropic or equivalent isotropic displacement parameters (Å^2^)

**Table:**

	*x*	*y*	*z*	*U*_iso_*/*U*_eq_	
Cl1	0.17726 (10)	0.35695 (7)	0.41886 (3)	0.05640 (19)	
O1	−0.1117 (2)	0.74773 (19)	0.00401 (9)	0.0527 (4)	
O2	0.3921 (2)	1.08846 (18)	−0.40006 (8)	0.0512 (4)	
N1	0.2890 (3)	0.73483 (19)	0.02965 (10)	0.0361 (3)	
C1	0.3419 (3)	1.0184 (2)	−0.30831 (11)	0.0352 (4)	
C2	0.1256 (3)	0.9169 (2)	−0.27999 (12)	0.0389 (4)	
H2	0.0280	0.9000	−0.3234	0.047*	
C3	0.0551 (3)	0.8416 (2)	−0.18864 (12)	0.0364 (4)	
H3	−0.0911	0.7753	−0.1706	0.044*	
C4	0.1999 (3)	0.8631 (2)	−0.12239 (11)	0.0319 (4)	
C5	0.4163 (3)	0.9639 (2)	−0.15134 (11)	0.0343 (4)	
H5	0.5157	0.9788	−0.1082	0.041*	
C6	0.4869 (3)	1.0425 (2)	−0.24313 (12)	0.0361 (4)	
H6	0.6309	1.1113	−0.2611	0.043*	
C7	0.1095 (3)	0.7782 (2)	−0.02462 (12)	0.0350 (4)	
C9	0.2519 (3)	0.6449 (2)	0.12294 (11)	0.0311 (3)	
C10	0.0413 (3)	0.6671 (2)	0.18553 (12)	0.0381 (4)	
H10	−0.0846	0.7411	0.1663	0.046*	
C11	0.0182 (3)	0.5794 (2)	0.27654 (12)	0.0391 (4)	
H11	−0.1230	0.5940	0.3186	0.047*	
C12	0.2064 (3)	0.4700 (2)	0.30466 (12)	0.0366 (4)	
C13	0.4176 (3)	0.4487 (2)	0.24347 (12)	0.0381 (4)	
H13	0.5441	0.3758	0.2632	0.046*	
C14	0.4404 (3)	0.5361 (2)	0.15282 (12)	0.0362 (4)	
H14	0.5830	0.5222	0.1113	0.043*	
C15	0.6246 (4)	1.1737 (3)	−0.43646 (14)	0.0596 (6)	
H15A	0.6329	1.2787	−0.4122	0.089*	
H15B	0.6418	1.2033	−0.5027	0.089*	
H15C	0.7567	1.0970	−0.4192	0.089*	
H1	0.435 (4)	0.741 (2)	0.0024 (12)	0.040 (5)*	

### Atomic displacement parameters (Å^2^)

**Table:**

	*U*^11^	*U*^22^	*U*^33^	*U*^12^	*U*^13^	*U*^23^
Cl1	0.0657 (4)	0.0623 (3)	0.0364 (3)	−0.0082 (2)	−0.0090 (2)	0.0036 (2)
O1	0.0295 (7)	0.0825 (10)	0.0405 (7)	−0.0090 (6)	−0.0035 (5)	0.0011 (7)
O2	0.0480 (8)	0.0676 (9)	0.0339 (7)	−0.0103 (6)	−0.0082 (6)	0.0022 (6)
N1	0.0262 (7)	0.0446 (8)	0.0345 (8)	−0.0009 (6)	−0.0015 (6)	−0.0026 (6)
C1	0.0342 (9)	0.0362 (9)	0.0347 (9)	0.0031 (7)	−0.0061 (7)	−0.0054 (7)
C2	0.0340 (9)	0.0461 (10)	0.0394 (9)	−0.0005 (7)	−0.0140 (7)	−0.0084 (8)
C3	0.0272 (8)	0.0396 (9)	0.0428 (10)	−0.0034 (7)	−0.0074 (7)	−0.0068 (7)
C4	0.0284 (8)	0.0315 (8)	0.0357 (9)	0.0027 (6)	−0.0049 (7)	−0.0067 (6)
C5	0.0306 (8)	0.0380 (9)	0.0359 (9)	−0.0012 (7)	−0.0096 (7)	−0.0077 (7)
C6	0.0303 (8)	0.0378 (9)	0.0391 (9)	−0.0056 (7)	−0.0052 (7)	−0.0047 (7)
C7	0.0297 (9)	0.0390 (9)	0.0361 (9)	−0.0016 (7)	−0.0046 (7)	−0.0070 (7)
C9	0.0283 (8)	0.0317 (8)	0.0331 (8)	−0.0041 (6)	−0.0052 (6)	−0.0051 (6)
C10	0.0299 (9)	0.0446 (10)	0.0400 (9)	0.0065 (7)	−0.0066 (7)	−0.0089 (7)
C11	0.0303 (8)	0.0501 (10)	0.0364 (9)	0.0006 (7)	0.0002 (7)	−0.0118 (8)
C12	0.0389 (9)	0.0373 (9)	0.0338 (9)	−0.0088 (7)	−0.0075 (7)	−0.0047 (7)
C13	0.0341 (9)	0.0357 (9)	0.0433 (10)	0.0025 (7)	−0.0093 (7)	−0.0026 (7)
C14	0.0271 (8)	0.0382 (9)	0.0413 (9)	−0.0002 (7)	−0.0006 (7)	−0.0072 (7)
C15	0.0558 (13)	0.0737 (14)	0.0414 (11)	−0.0162 (11)	−0.0041 (9)	0.0068 (10)

### Geometric parameters (Å, º)

**Table:**

Cl1—C12	1.7430 (17)	C5—H5	0.9300
O1—C7	1.222 (2)	C6—H6	0.9300
O2—C1	1.357 (2)	C9—C10	1.387 (2)
O2—C15	1.417 (2)	C9—C14	1.390 (2)
N1—C7	1.363 (2)	C10—C11	1.383 (2)
N1—C9	1.416 (2)	C10—H10	0.9300
N1—H1	0.831 (19)	C11—C12	1.383 (2)
C1—C6	1.388 (2)	C11—H11	0.9300
C1—C2	1.391 (2)	C12—C13	1.376 (2)
C2—C3	1.370 (2)	C13—C14	1.378 (2)
C2—H2	0.9300	C13—H13	0.9300
C3—C4	1.395 (2)	C14—H14	0.9300
C3—H3	0.9300	C15—H15A	0.9600
C4—C5	1.389 (2)	C15—H15B	0.9600
C4—C7	1.488 (2)	C15—H15C	0.9600
C5—C6	1.383 (2)		
			
C1—O2—C15	118.93 (14)	C10—C9—C14	119.44 (15)
C7—N1—C9	126.38 (14)	C10—C9—N1	122.65 (14)
C7—N1—H1	116.3 (13)	C14—C9—N1	117.86 (14)
C9—N1—H1	115.8 (13)	C11—C10—C9	120.10 (15)
O2—C1—C6	125.11 (15)	C11—C10—H10	120.0
O2—C1—C2	115.52 (14)	C9—C10—H10	120.0
C6—C1—C2	119.37 (15)	C12—C11—C10	119.59 (16)
C3—C2—C1	120.48 (15)	C12—C11—H11	120.2
C3—C2—H2	119.8	C10—C11—H11	120.2
C1—C2—H2	119.8	C13—C12—C11	120.83 (16)
C2—C3—C4	120.88 (15)	C13—C12—Cl1	119.26 (13)
C2—C3—H3	119.6	C11—C12—Cl1	119.91 (14)
C4—C3—H3	119.6	C12—C13—C14	119.51 (15)
C5—C4—C3	118.26 (15)	C12—C13—H13	120.2
C5—C4—C7	124.18 (14)	C14—C13—H13	120.2
C3—C4—C7	117.56 (14)	C13—C14—C9	120.52 (15)
C6—C5—C4	121.25 (15)	C13—C14—H14	119.7
C6—C5—H5	119.4	C9—C14—H14	119.7
C4—C5—H5	119.4	O2—C15—H15A	109.5
C5—C6—C1	119.75 (15)	O2—C15—H15B	109.5
C5—C6—H6	120.1	H15A—C15—H15B	109.5
C1—C6—H6	120.1	O2—C15—H15C	109.5
O1—C7—N1	122.75 (16)	H15A—C15—H15C	109.5
O1—C7—C4	121.52 (15)	H15B—C15—H15C	109.5
N1—C7—C4	115.72 (14)		
			
C15—O2—C1—C6	9.0 (3)	C3—C4—C7—O1	−26.4 (2)
C15—O2—C1—C2	−171.69 (17)	C5—C4—C7—N1	−28.3 (2)
O2—C1—C2—C3	−179.17 (15)	C3—C4—C7—N1	152.68 (15)
C6—C1—C2—C3	0.2 (3)	C7—N1—C9—C10	−35.0 (3)
C1—C2—C3—C4	−0.8 (3)	C7—N1—C9—C14	147.70 (17)
C2—C3—C4—C5	0.5 (2)	C14—C9—C10—C11	−0.9 (2)
C2—C3—C4—C7	179.57 (15)	N1—C9—C10—C11	−178.21 (15)
C3—C4—C5—C6	0.5 (2)	C9—C10—C11—C12	0.1 (3)
C7—C4—C5—C6	−178.52 (15)	C10—C11—C12—C13	0.7 (3)
C4—C5—C6—C1	−1.1 (3)	C10—C11—C12—Cl1	−179.24 (13)
O2—C1—C6—C5	−179.93 (15)	C11—C12—C13—C14	−0.7 (3)
C2—C1—C6—C5	0.8 (2)	Cl1—C12—C13—C14	179.24 (13)
C9—N1—C7—O1	2.7 (3)	C12—C13—C14—C9	−0.1 (3)
C9—N1—C7—C4	−176.35 (15)	C10—C9—C14—C13	0.9 (2)
C5—C4—C7—O1	152.67 (17)	N1—C9—C14—C13	178.35 (15)

### Hydrogen-bond geometry (Å, º)

**Table:**

*D*—H···*A*	*D*—H	H···*A*	*D*···*A*	*D*—H···*A*
N1—H1···O1^i^	0.83 (4)	2.47 (2)	3.222 (2)	151 (2)
C14—H14···O1^i^	0.93	2.56	3.251 (2)	131

Symmetry code: (i) *x*+1, *y*, *z*.
